# Supplementary material for: Learning curve and surgical outcome of robotic assisted colorectal surgery with ERAS program
Source: Sci Rep. 2022 Nov 29;12:20566. doi: 10.1038/s41598-022-24665-w (PMC9709162; doi:10.1038/s41598-022-24665-w)
Supplement: Supplementary file 2 — Supplementary Information 2. [file 41598_2022_24665_MOESM2_ESM.zip › RAL Raw data-Ñ[▒K/106/OP N_ñw╝╨░O▒Kñσ.pdf]

W52 / W52

臺中榮民總醫院
手術室護理記錄

[redacted]

基本資料

- 室別： 開刀房-17室，手術科別： CRS，手術類別： 常規，日期： 2021/08/19
- 進入時間： 2000 ； 麻醉開始時間： 2005 ； Time Out時間： 2135 ； 劃刀時間： 2136 ； 手術結束時間： 0200 送出時間： 0230 ；
- 麻醉方式： GE

  診斷：A COLON C WITH PARTIAL OBSTRUCTION

  手術名稱：Robot Assisted Colectomy(R)

  手術局部用藥：NIL

  術前預防性抗生素：
  - 抗生素名稱一： Veterin 1GM IVD由麻醉護理師給予
  - 時間： 2120
  - 上次簽章: 邱欐婇

  ，追加預防性抗生素：
- 手術醫師： 蔣鋒帆
- 麻醉醫師： VS 劉恆豪
-
  - 刷手護士A ： 陳妍希／陳佳琪，時間： 2000，接替刷手一： 林愛甯，時間： 2200，接替刷手二： 邱欐婇，時間： 2300
  - 流動護士A ： 邱欐婇，時間： 2000，接替流動一： 王筱瑜，時間： 2300，接替流動二： 林怡廷，時間： 0000
- 更多接替及流動護士： 接替刷手三： 林書綾，時間： 0100

皮膚狀況

A-V Shunt： 無

術前： 完整

記錄者： 上次簽章: 邱欐婇

術後： 不完整

- 6.水泡

部位(術後)：

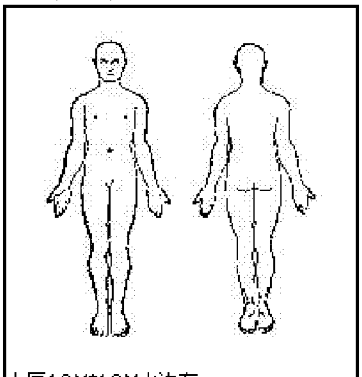

上唇1CM*1CM水泡存

記錄者： 林怡廷

手術準備

手術臥式 單一手術臥式

-
  - 手術臥式： 仰臥
  - 使用附件： 溫毯 38 ℃，頭圈，其它： 頭架+海綿塊*4
  - 皮膚準備： 2% Chlorhexidine
  - 電燒： 使用電燒: 電極片位置： 右小腿，記錄者： 上次簽章: 邱欐婇

止血帶T： 無

植入物

無

標本送檢

- 細菌培養 無
- 細胞學 無
- 冰凍切片 無
- 病理標本
  組織名稱一：A-COLON
  ,
  組織名稱二：appendix
- 截肢 無

標本交付 標本： 2 袋，標本： 0 盒，標本： 0 瓶，細胞學： 0 管，Culture Tube： 0 管，被交付醫師： Fellow 陳映菁，

- 記錄者： 上次簽章: 林怡廷
- 時間： 0150

傷口術後敷料

傷口敷料： 傷口敷料： 無

- 請於圖上標示部位：

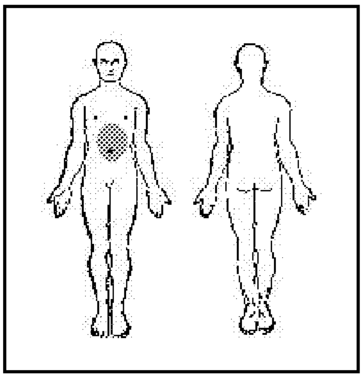

- 傷口敷料： 紗布

導管

導管	天數	導管資訊	插入日期	插入時間	更換日期	拔除日期	拔除時間	拔除原因	預計拔除/更換	預計拔除/更換日期
A-line (1)	2	號碼:20 G; 部位:右手;	2021/08/19	2100						
Foley(1)	2	號碼:16 Fr.; 部位:膀胱;	2021/08/19	2110						
Epidural PCA(1)	2	部位:硬脊膜;	2021/08/19	2000						
Double Lumen CVC(1)	2	號碼:8 Fr.; 深度:15CM; 部位:subclavian (右); 象限:RU;	2021/08/19	2110						
Penrose Drain(1)	1	號碼:12 mm.; 部位:肝下;	2021/08/20	0130						

用物計數

- 手術中出血量： 100 c.c.
- 手術中尿量： 350 c.c.
-
  - 其它： 無

用物計數： 多切口同時縫合

- 1.器械： 正確，2.縫針： 正確，3.刀片： 正確，4.紗布： 正確，5.腹部墊： 未使用，6.紗球： 未使用，7.PEANUTS ： 未使用，8.紗條： 未使用，9.絲帶： 未使用，10.針頭： 未使用，11.Vessel loop： 未使用，12.Boots： 未使用，13.Jelco： 未使用，14.棉條： 未使用，
  - 第一次：
    - 刷手護士 ： 林書綾
    - 流動護士： 上次簽章: 林怡廷

- 時間： 0130

,

- 第二次：
  - 刷手護士 ： 林書綾
  - 流動護士： 上次簽章: 林怡廷
  - 時間： 0140

,

- 第三次：
  - 刷手護士 ： 林書綾
  - 流動護士： 上次簽章: 林怡廷
  - 時間： 0150

備註

備註：NIL

- 記錄者： 上次簽章: 林怡廷
- 時間： 0020

填寫護理人員 林怡廷

正式摘要

W52 / W52

doctorRank:B
